# Intraoperative abobotulinumtoxinA alleviates pain after surgery and improves general wellness in a translational animal model

**DOI:** 10.1038/s41598-022-25002-x

**Published:** 2022-12-13

**Authors:** Sylvie Cornet, Denis Carré, Lorenzo Limana, David Castel, Sigal Meilin, Ron Horne, Laurent Pons, Steven Evans, Stephane Lezmi, Mikhail Kalinichev

**Affiliations:** 1 Ipsen Innovation, Les Ulis, France; 2 Ipsen Bioinnovation, Abingdon, UK; 3 MD Biosciences, Ness Ziona, Israel; 4 Present Address: Addex Therapeutics, Geneva, Switzerland; 5 Present Address: Excilone Sercives, Jouy en Josas, France

**Keywords:** Preclinical research, Experimental models of disease

## Abstract

Pain after surgery remains a significant healthcare challenge. Here, abobotulinumtoxinA (aboBoNT-A, DYSPORT) was assessed in a post-surgical pain model in pigs. Full-skin-muscle incision and retraction surgery on the lower back was followed by intradermal injections of either aboBoNT-A (100, 200, or 400 U/pig), vehicle (saline), or wound infiltration of extended-release bupivacaine. We assessed mechanical sensitivity, distress behaviors, latency to approach the investigator, and wound inflammation/healing for 5–6 days post-surgery. We followed with immunohistochemical analyses of total and cleaved synaptosomal-associated protein 25 kD (SNAP25), glial fibrillary acidic protein (GFAP), ionized calcium-binding adaptor protein-1(Iba1), calcitonin gene-related peptide (CGRP) and substance P (SP) in the skin, dorsal root ganglia (DRG) and the spinal cord of 400 U aboBoNT-A- and saline-treated animals. At Day 1, partial reversal of mechanical allodynia in aboBoNT-A groups was followed by a full reversal from Day 3. Reduced distress and normalized approaching responses were observed with aboBoNT-A from 6 h post-surgery. Bupivacaine reversed mechanical allodynia for 24 h after surgery but did not affect distress or approaching responses. In aboBoNT-A-treated animals cleaved SNAP25 was absent in the skin and DRG, but present in the ipsilateral dorsal horn of the spinal cord. In aboBoNT-A- versus saline-treated animals there were significant reductions in GFAP and Iba1 in the spinal cord, but no changes in CGRP and SP. Analgesic efficacy of aboBoNT-A appears to be mediated by its activity on spinal neurons, microglia and astrocytes. Clinical investigation to support the use of aboBoNT-A as an analgesic drug for post-surgical pain, is warranted.

## Introduction

Pain after surgery remains a significant healthcare challenge as a significant number of patients continue to suffer from moderate to severe pain, some of them for a prolonged period^1,2^. Pain after surgery can be accompanied by anxiety, depression, elevated stress, and catastrophizing, which can enhance acute pain after surgery, facilitate its chronicity and interfere with postoperative recovery and rehabilitation. The management of pain after surgery, especially severe pain, continues to rely heavily on opioid drugs, despite associated acute side-effects, slower healing and risk of addiction^3^. There is an urgent need for novel, non-opioid, analgesic drugs that are well-tolerated and provide effective, prolonged relief from pain after surgery.

Botulinum toxins (BoNTs) are a diverse group of proteins produced by *Clostridium botulinum* and related bacteria^4^. In nature, they are synthesized as a single-chain polypeptide that is modified post-translationally to form two polypeptide chains, a 100-kDa heavy chain (HC) and a 50-kDa light chain (LC), which are connected by a disulfide bond^5^. Upon binding to cell-surface receptors, the molecule is internalized via an endosome, followed by translocation of the LC into the cytosol. The LC cleaves intracellular transport proteins known as SNARE proteins and inhibits cellular secretion from the target cell, to impart inhibitory effects on synaptic transmission^5^. For several decades, BoNT type A (BoNT-A) preparations have been used in esthetic dermatology as well as therapeutic applications, providing effective and long-lasting relief for patients with muscle hyperactivity disorders. Recently, BoNT-A has been approved for chronic migraine and there is an accumulated evidence on its efficacy in a range of pain conditions in the clinic and in animal models^6,7^. Here, we evaluated analgesic activity of abobotulinumtoxinA (aboBoNT-A; DYSPORT) in a model of post-surgical pain in domestic pigs^8^.

Post-surgical pain in pigs^8^ is a translational animal model based on several considerations. Firstly, there is a remarkable similarity between the pig and the human skin in terms of its structure, innervation, immune response and post-incisional healing^9,10^. Behavioral changes seen in pigs following full-skin and muscle incision and retraction (SMIR) surgery, that mimics operations performed in the clinic, are relevant to evoked pain, non-evoked, resting pain and pain-related anxiety- and depression-like reactivity^8,11^. Mechanical hypersensitivity and guarding behavior are considered surrogates for evoked, movement-related pain and non-evoked, resting pain, respectively, which are seen in both human patients after surgery^12,13^ and in a rodent model^14^. Reduced social interaction, withdrawal when approached as well as reluctance to initiate approach of a caregiver, on the other hand, can be considered as surrogates for post-surgical pain-mediated anxiety or depression seen in human patients^15^ and in rodent models of post-surgical pain^16,17^.

We hypothesized that intraoperative aboBoNT-A treatment would be well-tolerated and provide effective and prolonged relief from post-SMIR surgical pain compared with its vehicle (saline) or infiltrated liposomal bupivacaine (EXPAREL; an approved, slow-release anesthetic). We used liposomal bupivacaine as a positive control in this study based on its effective pain relief, lasting up to 72 h, in a range of human surgical conditions^18^. Also, previously we have shown analgesic efficacy of liposomal bupivacaine in the current model of post-surgical pain^19^.

## Results

### von Frey test

**Figure 1:**
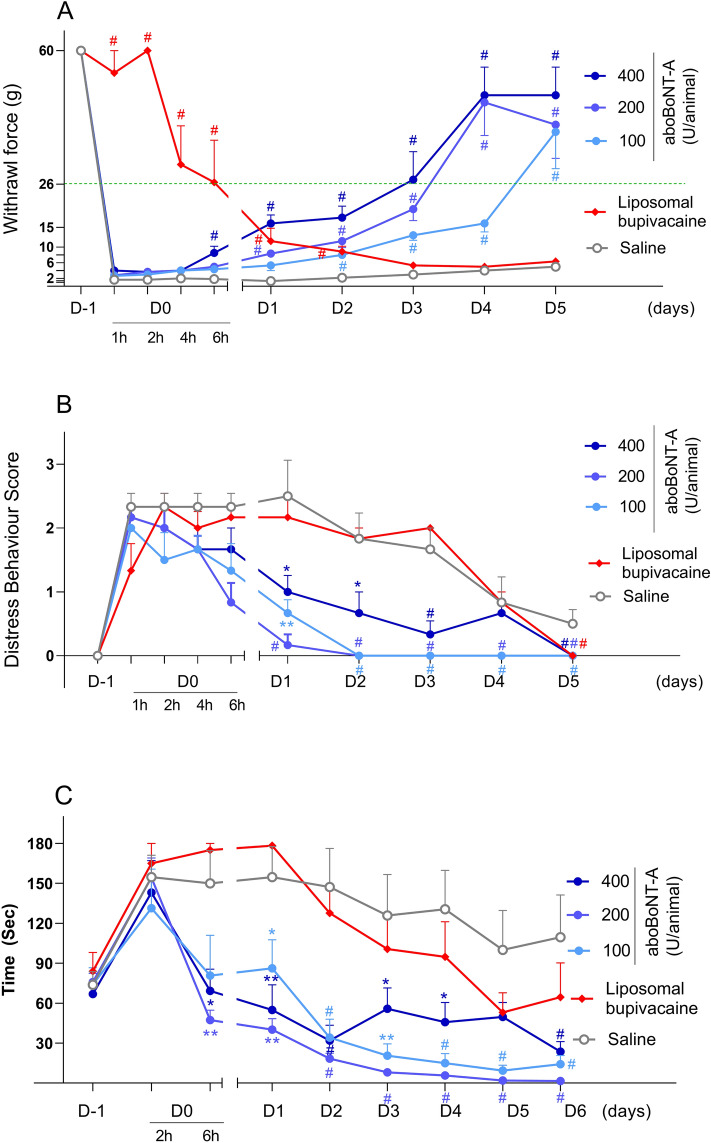
Effects of aboBoNT-A, saline, and liposomal bupivacaine on postsurgical pain-related behavioral responses in pigs. Mechanical sensitivity/withdrawal force (WF; g, **A**), DBS (**B**) and approaching time (sec; **C**) in animals that received a full-skin and muscle incision and retraction surgery (Day 0), followed by intraoperative treatment with either aboBoNT-A (100 U, 200 U, or 400 U), saline, or liposomal bupivacaine (n = 6/group). The green dotted line in panel (**A**) at 26 g represents the mechanical sensitivity threshold between normal mechanical sensitivity and mechanical hypersensitivity/allodynia. Each point represents the observed mean (± SEM). *p < 0.05, **p < 0.01, ^#^p < 0.001 vs saline-injected animals. AboBoNT-A, abobotulinumtoxinA; DBS, distress behavior score; U, unit; WF, withdrawal force.

At baseline, one day before surgery (Day -1), all animals responded to the withdrawal force (WF) of 60 g. Therefore, none were excluded from the study. One hour following surgery (Day 0), WF of the saline group decreased to 1 to 2 g and remained low throughout 5 days following surgery, showing only slow and minimal recovery to 5.0 ± 0.86 g on Day 5 (Fig. 1A).

AboBoNT-A increased WF dose-dependently in comparison to saline treatment (Fig. 1A). Specifically, animals injected with 400 U aboBoNT-A showed a marked and significant increase in WF on Day 1, followed by similar effects seen in the 200 U and 100 U aboBoNT-A groups from Days 2 and 3, respectively (Fig. 1A). A full recovery to baseline sensitivity (60 g) in response to aboBoNT-A treatment was reached 4 to 5 days after injection (Fig. 1A). In liposomal bupivacaine-treated animals a full reversal of mechanical allodynia up to 2 h post-administration was followed by a partial reversal 4 and 6 h post-administration. From Day 1, the WF of bupivacaine-injected animals continued to decline and starting Day 3 it was similar to that of saline-treated controls (Fig. 1A).

**Figure 2:**
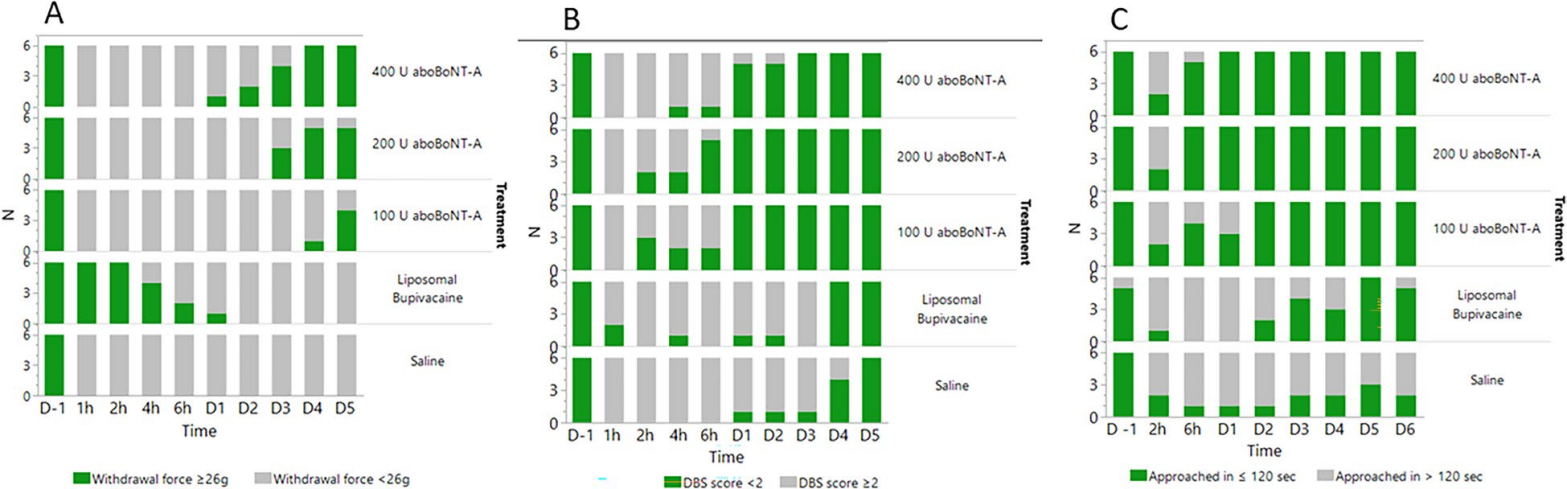
Effects of aboBoNT-A, saline, and liposomal bupivacaine on the number of animals exhibiting postsurgical pain-related versus normal responses in each group. Mechanical sensitivity/withdrawal force (**A**), DBS (**B**) and approaching time (**C**) in animals that received a full-skin and muscle incision and retraction surgery (Day 0), followed by intraoperative treatment with either aboBoNT-A (100 U, 200 U, or 400 U), saline or liposomal bupivacaine (n = 6/group). Panel (**A**) shows the number of animals (N) with normal mechanical sensitivity (WF ≥ 26 g) vs hypersensitivity/allodynia (WF < 26 g). Panel (**B**) shows the number of animals exhibiting moderate to high distress (DBS ≥ 2) vs those with minimal or no distress (DBS < 2). Panel (**C**) shows the number of animals that initiated approach in ≤ 120 s or those that took more than 120 s in the Approaching test. AboBoNT-A, abobotulinumtoxinA; DBS, distress behavior score; U, unit.

The number of animals responding to WF of ≥ 26 g (considered as a normal mechanical sensitivity in intact animals) was significantly influenced by treatment. Specifically, none of saline-treated animals reached this threshold at any time-point after surgery. In contrast, in the aboBoNT-A 400 U group, while only one animal showed this response on Day 1, all 6 animals in this group did so on Days 4 and 5 (Fig. 2A). In the aboBoNT-A 200 U group, three, five and five animals showed normal mechanical sensitivity on Days 3, 4 and 5, respectively. In the aboBoNT-A 100 U group, only one animal showed these responses on Day 4, whereas four animals responded so by Day 5 (Fig. 2A). The opposite pattern of activity was seen in liposomal bupivacaine-injected animals. Specifically, while WF responses of all 6 animals were ≥ 26 g 1 and 2 h after the surgery, only four and two animals showed these responses 4 and 6 h after surgery, respectively (Fig. 2A). In the liposomal bupivacaine group, WF ≥ 26 g was detected in one animal only on Day 1, while none of these animals showed normal mechanical sensitivity on Days 2–5 (Fig. 2A).

### DBS test

At baseline, one day before surgery (Day -1), intact animals were distress-free (i.e. DBS score 0; Fig. 1B). After surgery, however, distress behaviors emerged as animals began exhibiting guarding behavior protecting the site of the incision and moving away when approached by the investigator (category scorings 3 and 4; see Materials and Methods). The DBS of the saline-treated animals remained high up to Day 3 after surgery, followed by a gradual decline on Days 4 and 5 (Fig. 1B). Animals injected with aboBoNT-A showed significant and sustained reductions in DBS when compared with the saline-treated controls starting Day 1, albeit without a clear dose-dependency (Fig. 1B). In contrast to aboBoNT-A, liposomal bupivacaine lacked efficacy in the DBS test throughout the study (Fig. 1B).

While one day before surgery all intact animals were distress-free (i.e. DBS scores of < 2), 1 h after surgery, distress scores of ≥ 2 predominated in nearly all animals (Fig. 2B). Between 2 and 6 h after surgery, there was a steady increase in the number of animals with no distress in groups treated with aboBoNT-A, but not in groups treated with saline or liposomal bupivacaine (Fig. 2B). Starting Day 1, behavior of virtually every animal treated with aboBoNT-A was distress-free, while on Days 1–3, the majority of saline- and liposomal bupivacaine-treated animals continued exhibiting distress (≥ 2; Fig. 2B). On Day 5 all animals were distress-free (Fig. 2B).

### Approaching test

At baseline, one day before surgery (Day-1), intact animals were quick to approach the investigator (the total mean 75 ± 4.57 s; Fig. 1C). Two hours after surgery, however, approaching time increased markedly and similarly across groups to greater than 140 s (Fig. 1C). Vehicle-treated animals remained slow to approach the investigator throughout the study (Fig. 1C). Animals treated with aboBoNT-A, however, showed a rapid normalization of approaching behavior. Specifically, at 6 h, latencies to approach the investigator returned to baseline values in all aboBoNT-A groups (Fig. 1C). Starting Day 1, latencies to approach continued to decline in animals treated with aboBoNT-A 100 U and 200 U and to a lesser degree in those treated with aboBoNT-A 400 U (Fig. 1C). In contrast to aboBoNT-A treatment, liposomal bupivacaine-treated animals continued exhibiting long latencies to approach the investigator up to Day 2, that was followed with a slow recovery to baseline responses by Day 5. Approaching latencies of liposomal bupivacaine-treated animals were largely similar to those of saline-treated controls across the study (Fig. 1C).

At baseline (Day -1), all animals except one, approached the investigator within 120 s (Fig. 2C). While there was a significant reduction in responders 2 h post-surgery across all groups, a clear effect of aboBoNT-A on approaching responses were noticed starting 6 h post-administration (Fig. 2C). Specifically, 6 h post-surgery, nearly all animals injected with aboBoNT-A 200 U and 400 U approached the investigator in less than 120 s (Fig. 2C). Starting Day 3, all animals treated with aboBoNT-A approached the investigator in less than 120 s (Fig. 2C). In contrast, only 1 vehicle-treated animal responded to the investigator in less than 120 s up to Day 2, while the number of responders increased gradually to 3 animals by the end of the study (Fig. 2C). Among liposomal bupivacaine-treated animals none approached the investigator in less than 120 s 6 h post-surgery and on Day 1 (Fig. 2C). Starting Day 2, there was a slow increase in the number of responders from 2 (Day 2), 4 (Day 3) and 5-6 animals (Days 5–6; Fig. 2C).

### Open field test

Locomotor activity in the Open field test was similar at Day 3 across all treatment groups for total distance travelled, the speed of locomotion and as the percentage of time spent in the central zone (Fig. S1 A–C). In the latter measure, there was a trend of a higher proportion of time spent in the center by animals injected with aboBoNT-A 400 U compared with other groups (Fig. S1 C).

### Body weight and clinical signs

All groups showed a comparable body weight growth during the study. There was no difference in absolute body weight as well as in percent body weight gain across aboBoNT-A-, saline-, and liposomal bupivacaine-injected groups on Days -5, 0 and 5 (Table S1). Similarly, there were no signs of limb paralysis or those of systemic toxicity in any of the aboBoNT-A-injected animals.

### Wound inflammation

Daily clinical evaluation of wounds showed that inflammation-related swelling was absent in all animals, whereas redness was rare and overall mild. There was no difference in redness score across groups (Fig. S2).

### Histopathology and IHC analyses

H&E examination of the skin cross-section samples, collected on Day 6, revealed that wound healing and inflammation were similar in aboBoNT-A- and saline-treated animals (Fig. S3). In addition, image analyses of CD31 IHC from the dermis around the incision area revealed similarity in blood micro vessel density in aboBoNT-A- and saline-treated animals (Fig. S4 A–C). The similarity in blood vessel size distribution in aboBoNT-A and saline-treated animals was confirmed by a quantitative analysis (Table S2).

**Figure 3:**
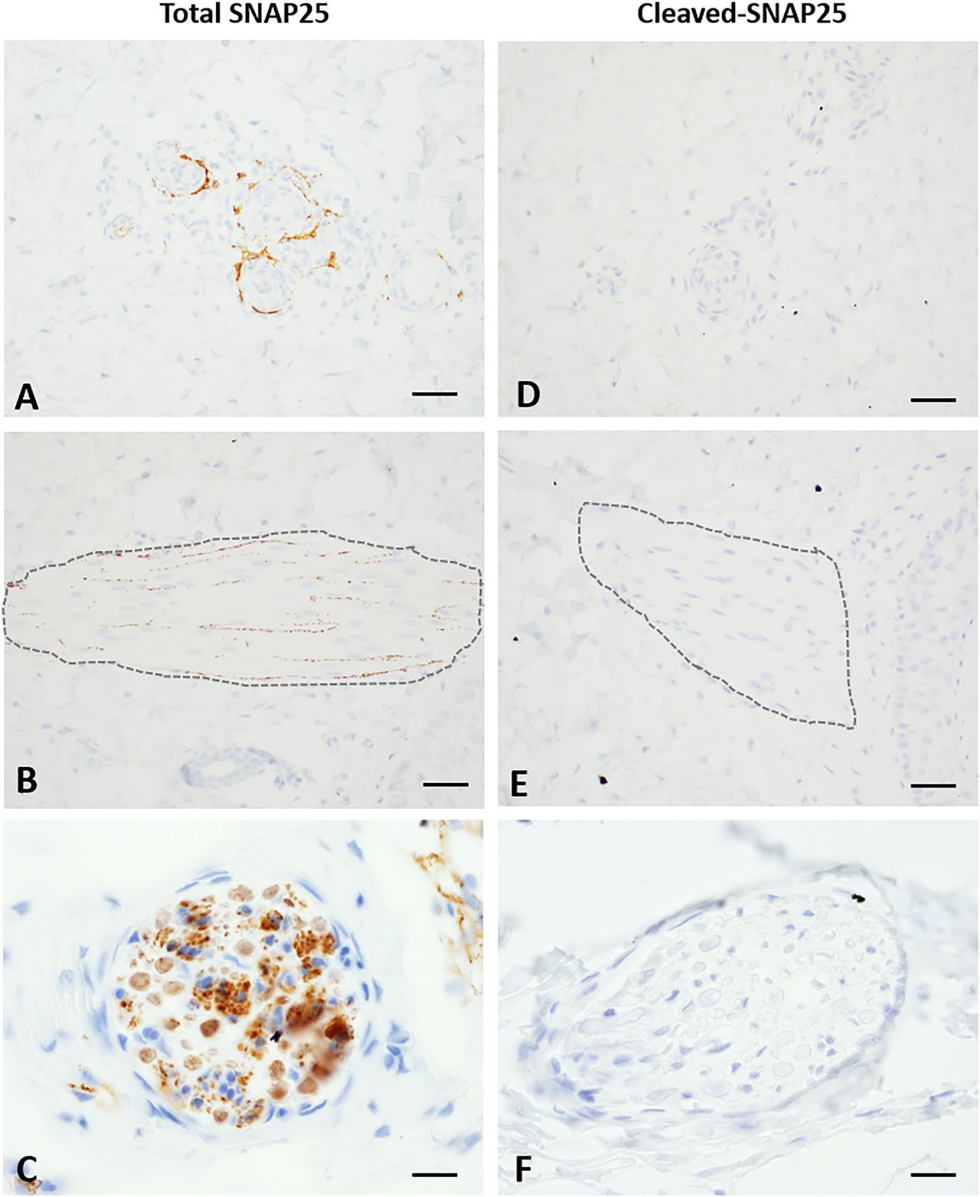
Presence of the total SNAP25 and absence of cleaved SNAP25 immunolabelling in the skin of aboBoNT-A-treated animals. Photomicrographs depict the total SNAP25 immunolabelling in small arteries (**A**), arrector pili muscles (**B**), and in nerve fibers of the dermis (**C**) in the skin of a representative animal that received a full-skin and muscle incision and retraction surgery (Day 0), followed by intraoperative treatment with aboBoNT-A (400 U) and tissue collection on Day 6. Cleaved SNAP25 immunolabelling was absent in corresponding areas (**D–F**) in the skin of a representative animal treated with aboBoNT-A (400 U). Scale bars: 50 µm (**A, B, D, E**); 25 µm (**C, F**). AboBoNT-A, abobotulinumtoxinA; U, unit.

In both aboBoNT-A- and saline-treated animals the total SNAP25 immunolabelling (N-terminal part) was detected in the skin adjacent to the injection site (Fig. 3). Specifically, the total SNAP25 immunolabelling was detected in nerve endings around small arteries, in arrector pili muscles and in nerves in the deep and superficial dermis (Fig. 3A–C). In contrast, the same area of the skin lacked cleaved SNAP25 in both aboBoNT-A- and saline-treated animals (Fig. 3D–F).

**Figure 4:**
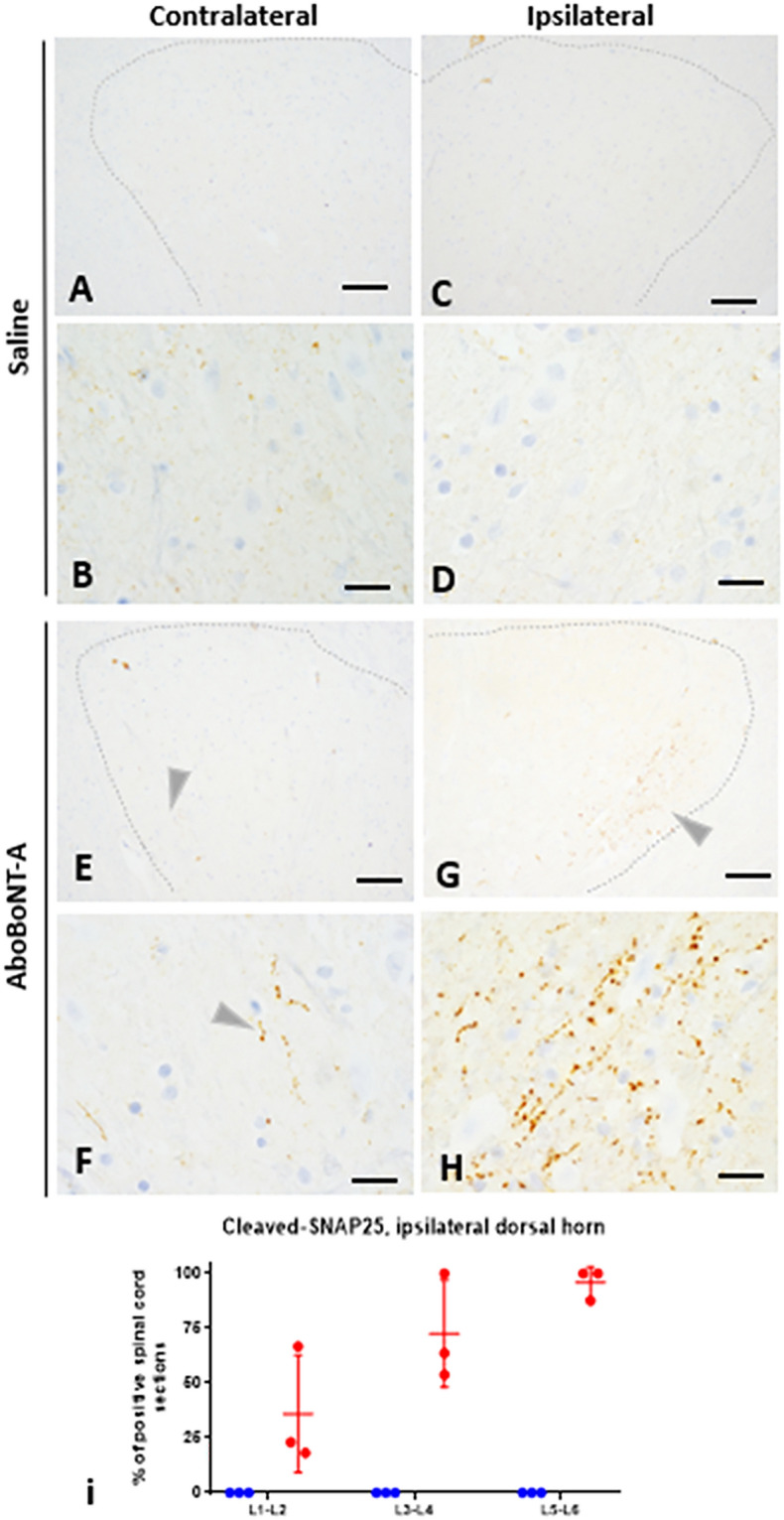
Assessment of cleaved SNAP25 immunolabelling in the spinal cord of saline- and aboBoNT-A treated animals. Photomicrographs depict lack of cleaved SNAP25 immunolabeling in the dorsal horn of the lumbar spinal cord contralateral  (**A, B**) and ipsilateral (**C, D**) to the incision (see text) in a representative animal that received a full-skin and muscle incision and retraction surgery (Day 0), followed by intraoperative treatment with saline. Also, photomicrographs depict presence of cleaved SNAP25 immunolabeling in the dorsal horn of the lumbar spinal cord ipsilateral (**G, H**) and contralateral (**E, F**) to the incision in a representative animal that received a full-skin and muscle incision and retraction surgery (Day 0), followed by intraoperative treatment with aboBoNT-A 400 U. In all aboBoNT-A-treated animals, cleaved SNAP25 was detected in the lateral part of the ipsilateral dorsal horn (**G**; arrowhead), corresponding to the substantia gelatinosa of Rolando. In the contralateral dorsal horn (**E, F**; arrowhead) a minimal staining was present in the same location. Panel I shows quantification of positive spinal cord sections for cleaved SNAP25 from lumbar (L) sections 1–2, 3–4, and 5–6 obtained from three representative animals treated with saline (blue symbols) and aboBoNT-A (red symbols), as well as their mean values (± SEM). Size bars: 100 µm (**A, C, E, G**) or 20 µm (**B, D, F, H**). AboBoNT-A, abobotulinumtoxinA; U, unit.

In vehicle-treated animals cleaved SNAP25 was absent throughout the lumbar spinal cord, including the contralateral (Fig. 4A, B) and ipsilateral dorsal horns (Fig. 4C, D). In contrast, we detected intense staining of cleaved SNAP25 in the lateral part of the dorsal horn, ipsilateral to the injection (Fig. 4G, H), while mild staining was detected in the lateral part of the dorsal horn contralateral to the injection (Fig. 4E, F). On both sides, the location of cleaved SNAP25 staining overlapped with the lateral part of the substantia gelatinosa of Rolando ^20^. Quantification of cleaved SNAP25 in the ipsilateral dorsal horn rostro-caudally in the lumbar spinal cord revealed its high intensity at the L5-L6 sections whereas milder staining was detected in more rostral sections (L1-L2 and L3-L4) of the lumbar spinal cord (Fig. 4I).

**Figure 5:**
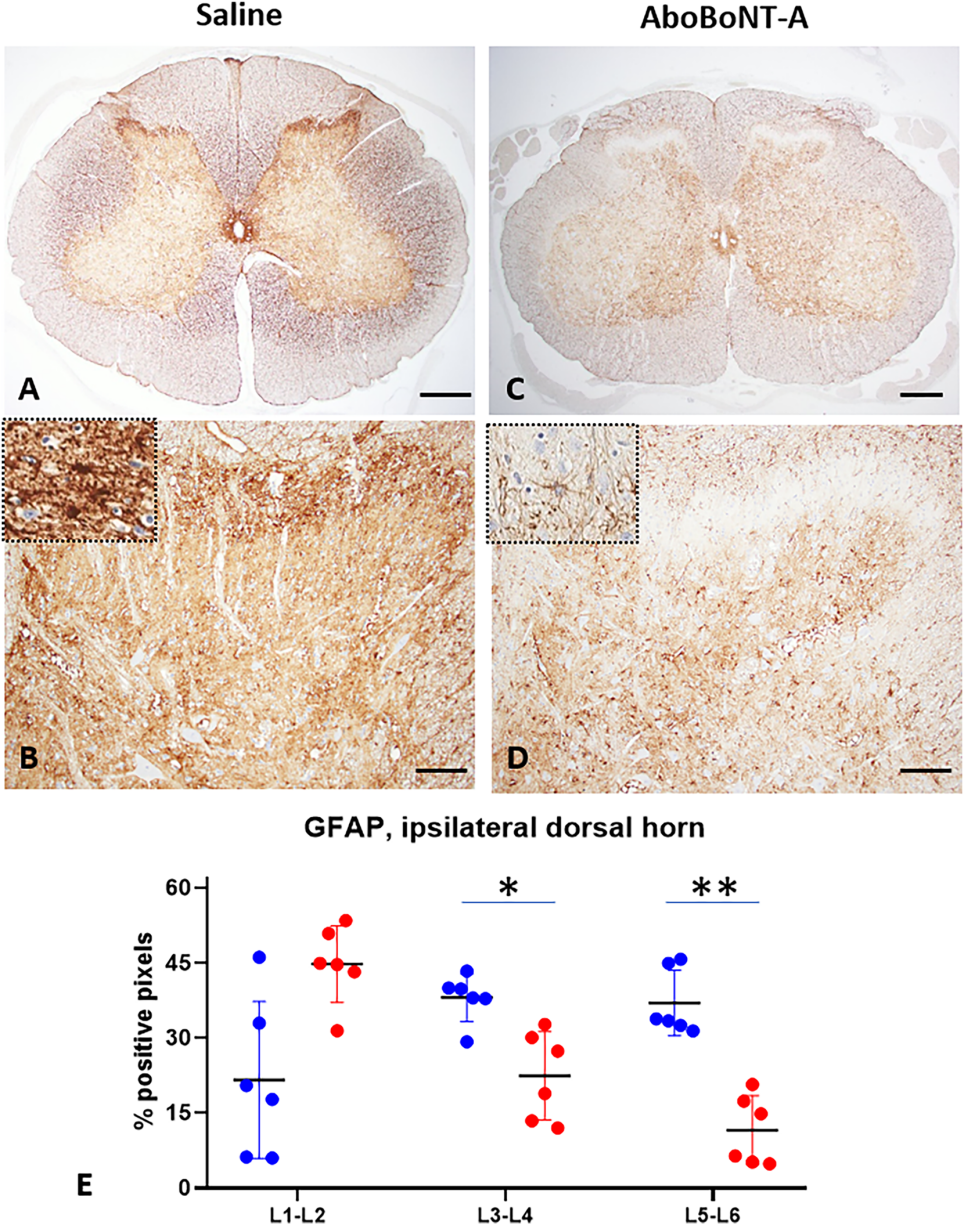
GFAP immunolabelling in the lumbar spinal cord of saline- and aboBoNT-A- treated animals. Photomicrographs are taken from L5-L6 sections of the lumbar spinal cord in representative animals that received a full-skin and muscle incision and retraction surgery (Day 0), followed by intraoperative treatment with either saline (**A, B**) or aboBoNT-A 400U (**C, D**) and tissue collection on Day 6. Scale bar: 1 mm (**A, C**), 200 µm (**B, D**). Insets in panels (**B**) and (**D**) depict GFAP stained astrocytes in saline- (**B**) and aboBoNT-A-treated (**D**) animals. Panel (**E**) shows quantification of GFAP immunolabelling in entire ipsilateral dorsal horns from lumbar (L) sections 1–2, 3–4, and 5–6 obtained from representative animals (n = 3/group) treated with either saline (blue symbols) or aboBoNT-A (red symbols; n = 2 sections per animal) as well as their mean values (± SEM). *p < 0.05, **p < 0.01 vs saline-injected animals. GFAP, glial fibrillary acidic protein; AboBoNT-A, abobotulinumtoxinA; U, unit.

We found a high level of GFAP immunostaining throughout the lumbar spinal cord of saline-treated animals (Fig. 5A,B). Specifically, GFAP staining was found to be diffuse and bilateral, with a particularly dense staining in the substantia gelatinosa of Rolando and around the central canal (Fig. 5A,B). In comparison to vehicle-treated controls, aboBoNT-A-treated animals showed a marked decrease in GFAP immunostaining which occurred diffusely and bilaterally (Fig. 5C,D). This reduction in GFAP immunostaining was particularly striking and similar in both ipsilateral and contralateral dorsal horns (substantia gelatinosa) as well as around the central canal of the spinal cord (Fig. 5C,D). Rostro-caudally, reductions of GFAP immunostaining in the lumbar spinal cord were observed in L3-L4 and L5-L6 sections (Fig. 5E).

**Figure 6:**
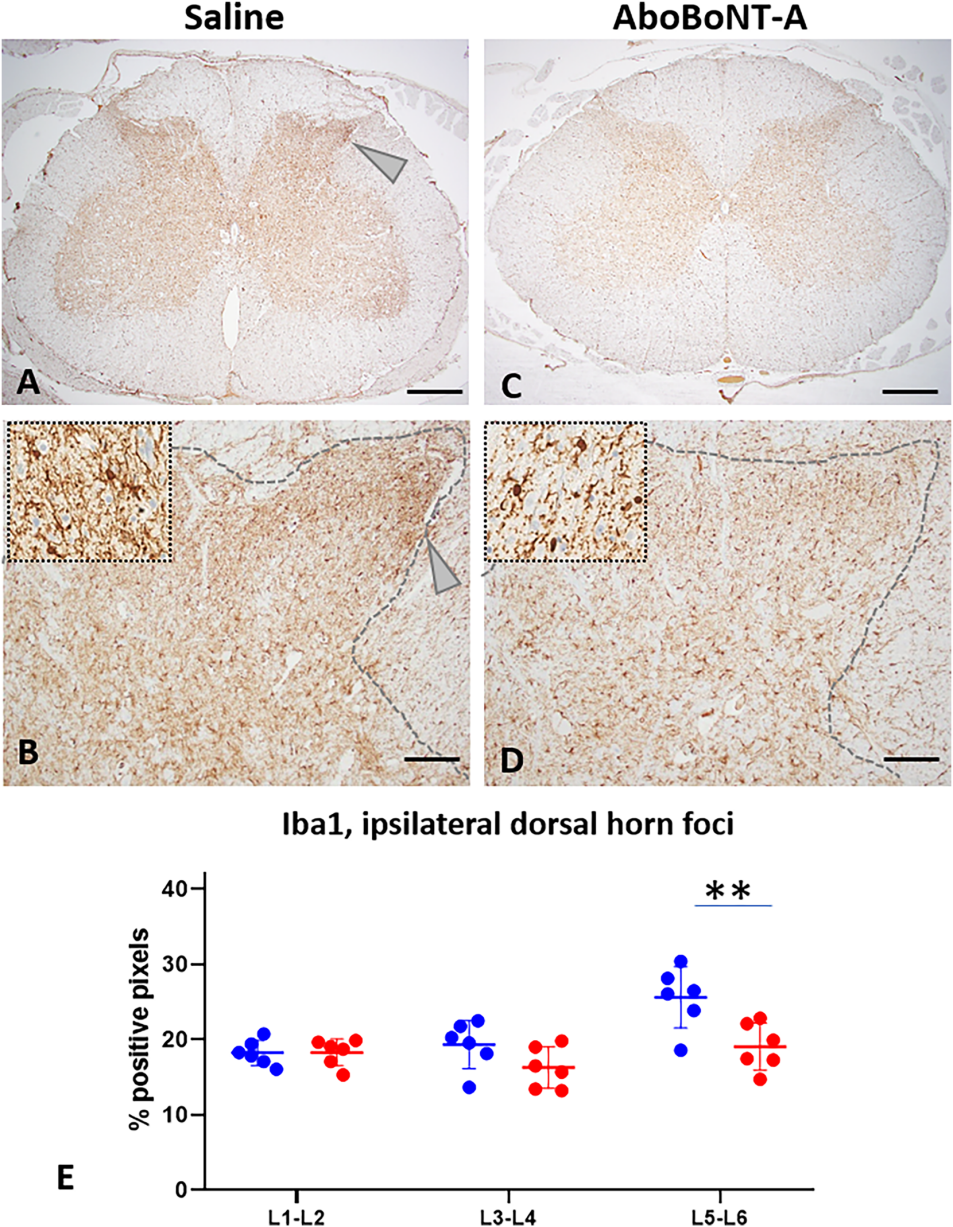
Iba1 immunolabelling in the lumbar spinal cord of saline- and aboBoNT-A- treated animals. Photomicrographs are taken from L5-L6 sections of representative animals that received a full-skin and muscle incision and retraction surgery (Day 0), followed by intraoperative treatment with either saline (**A, B**) or aboBoNT-A 400U (**C, D**) and tissue collection on Day 6. Insets in panels (**B**) and (**D**) depict Iba1 stained microglia in saline- (**B**) and aboBoNT-A-treated (**D**) animals. Panel (**E**) shows quantification of Iba1 immunolabelling in the foci with high intensity labelling (arrowhead) from lumbar (L) sections 1–2, 3–4, and 5–6 in representative animals (n = 3/group) treated with either saline (blue symbols) or aboBoNT-A (red symbols; n = 2 sections per animal) as well as their mean values (± SEM). Scale bar: 1 mm (**A, C**), 200 µm (**B, D**). **p < 0.01 vs saline-injected animals. Ionized calcium-binding adaptor protein-1, Iba1; AboBoNT-A, abobotulinumtoxinA; U, unit.

We found a high level of Iba1 immunolabelling in the grey matter of the lumbar spinal cord in saline-treated animals (Fig. 6A,B). Specifically, we found Iba1 immunolabelling to be diffuse and bilateral, with a high density staining in a focal point of the lateral part of the ipsilateral dorsal horn (substantia gelatinosa of Rolando) of the L5-L6 spinal cord sections (Fig. 6A,B, arrowheads). In comparison to vehicle-treated controls, aboBoNT-A-treated animals showed a moderate decrease in Iba1 immunolabelling which occurred diffusely and bilaterally (Fig. 6C,D). A focal point of dense Iba1 immunolabelling noted in vehicle-treated animals at the L5-L6 level was fully absent in aboBoNT-A animals (Fig. 6C,D). Moderate decrease in Iba1 immunolabelling in response to aboBoNT-A treatment was detected on L5-L6 sections of the spinal cord (Fig. 6E). At more rostral sections of the lumbar spinal cord (L1-L2 and L3-L4), Iba1 immunolabelling was similar in aboBoNT-A- and saline-treated animals (Fig. 6E).

**Figure 7:**
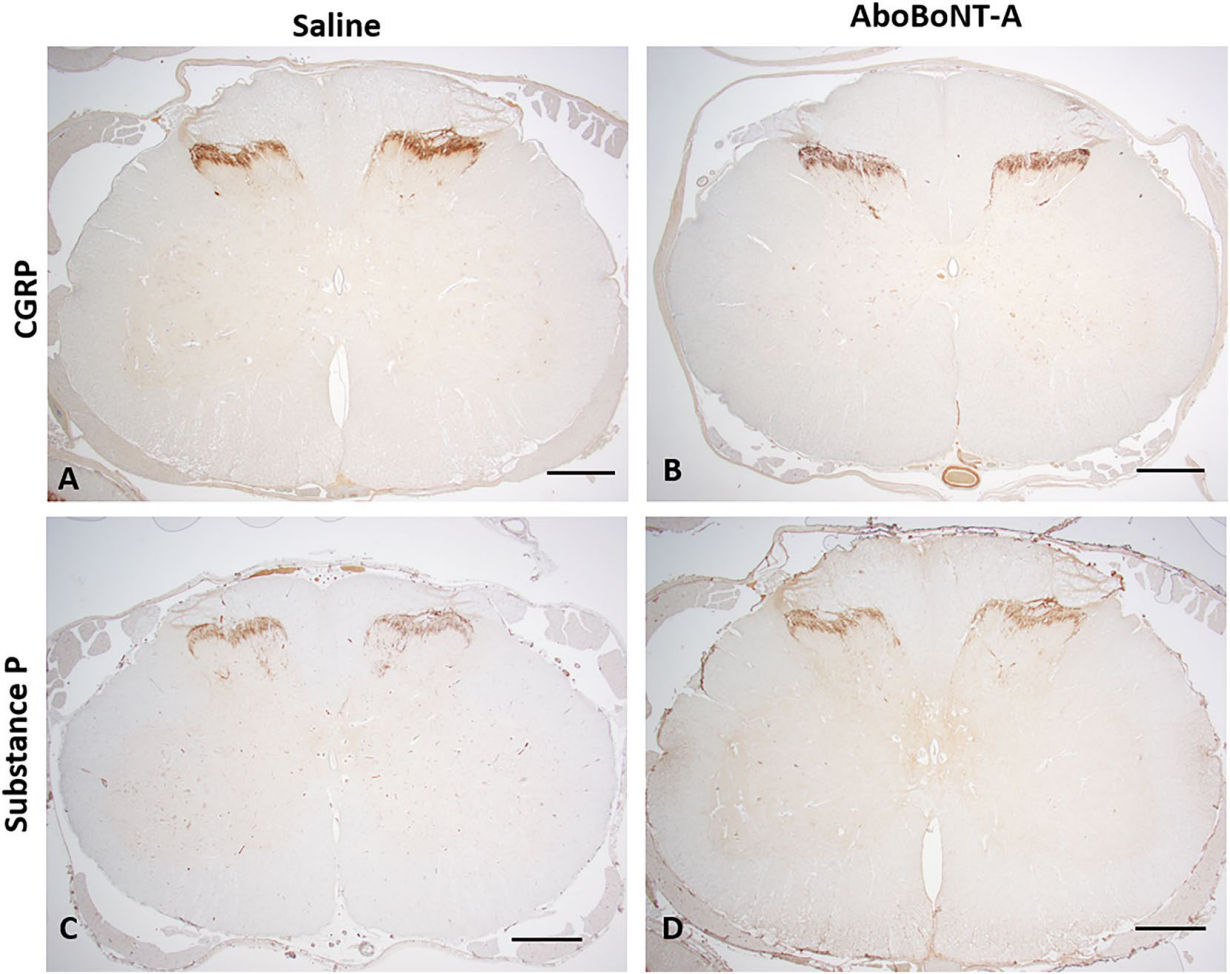
CGRP and SP immunolabelling in the lumbar spinal cord of saline- and aboBoNT-A-treated animals. Photomicrographs are taken from L5-L6 sections taken from representative animals that received a full-skin and muscle incision and retraction surgery (Day 0), followed by intraoperative treatment with either saline (**A, C**) or aboBoNT-A 400U (**B, D**) and tissue collection on Day 6. Scale bars: 1 mm. CGRP, calcitonin gene-related peptide; SP, Substance P; AboBoNT-A, abobotulinumtoxinA; U, unit.

We detected high level of CGRP immunolabelling bilaterally in the upper lamina of the dorsal horn of the spinal cord, largely overlapping with the location of the substantia gelatinosa of Rolando (Fig. 7A,B). The intensity of CGRP immunolabelling was similar in saline- and aboBoNT-A-treated animals (Fig. 7A,B). We also found a moderate level of SP immunolabelling bilaterally in upper lamina of the dorsal horn of the lumbar spinal cord, also in the substantia gelatinosa of Rolando (Fig. 7C,D). There was no difference in SP immunolabelling between aboBoNT-A and saline-treated animals in the dorsal horn (Fig. 7C,D).

**Figure 8:**
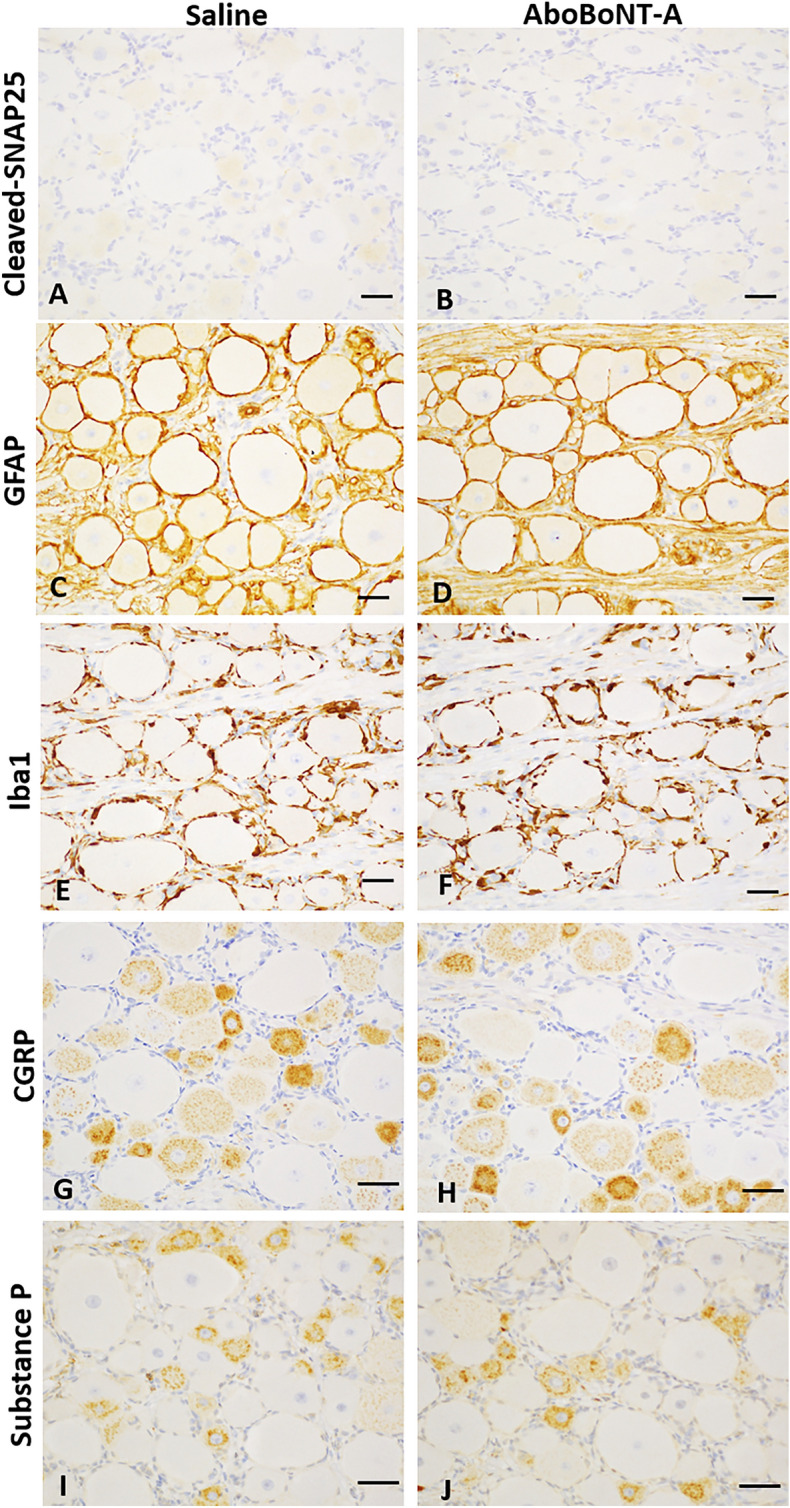
Cleaved SNAP25, GFAP, Iba1, CGRP, and SP immunolabelling in the DRGs of saline- and aboBoNT-A-treated animals. Photomicrographs are taken from DRGs located on L5-L6 sections of the lumbar spinal cord ipsilateral to the incision in representative animals that received a full-skin and muscle incision and retraction surgery (Day 0), followed by intraoperative treatment with either saline (**A, C, E, G, I**) or 400 U aboBoNT-A (**B, D, F, H, J**) and tissue collection on Day 6. Scale bars: 50 µm. GFAP, glial fibrillary acidic protein; CGRP, calcitonin gene-related peptide; SP, Substance P; Iba1, ionized calcium-binding adaptor protein-1; AboBoNT-A, abobotulinumtoxinA; U, unit.

In the DRG emerging form the L4-L5-L6 levels of the lumbar spinal cord we detected lack of cleaved SNAP25 in both saline- and aboBoNT-A-treated animals (Fig. 8A,B). We also detected intense GFAP, Iba1, CGRP and SP immunolabelling in the same DRGs (Fig. 8C–J). There was no difference in labelling of these markers between saline- and aboBoNT-A-treated animals (Fig. 8C–J).

## Discussion

This study demonstrated that intraoperative administration of aboBoNT-A provided effective and prolonged pain relief related to evoked pain, non-evoked pain and pain-associated anxiety- and depression-like reactivity in a translational, post-surgical domestic pig model. We saw a marked post-operative reduction of withdrawal force from 60 g in all healthy animals to less than 2 g in animals after surgery, indicative of allodynia (mechanical hypersensitivity). AboBoNT-A treatment was associated with dose- and time-dependent, enduring reversal of SMIR-induced allodynia from 24 h after administration. Specifically, a robust, ≥ 30% reversal of allodynia was seen from Days 1, 3 and 4 onwards in animals injected with aboBoNT-A 400 U, 200 U and 100 U, respectively. By Day 5, mechanical sensitivity of all aboBoNT-A-injected animals approached its pre-operative baseline (WF of 60 g). Furthermore, aboBoNT-A dose-dependency in mechanical sensitivity was also detected when assessing the number of animals with normal mechanical sensitivity (i.e. withdrawal responses of 26 g or above) compared with those with allodynia (i.e. withdrawal responses less than 26 g) per group. The results obtained in pigs confirm and expand an earlier evidence of the analgesic activity of BoNTs on evoked pain-related responses in rodent models of postsurgical pain. For example, an intraplantar administration of 3.5 and 7 U/kg, but not of 1 U/kg of onabotulinumtoxinA (onaBoNT-A) in rats resulted in similar, near-maximal and enduring (up to 10 days) reversal of mechanical hyperalgesia induced by the gastrocnemius muscle cut^21^. Also, onaBoNT-A given via intraplanar or intrathecal routes, 24 or 48 h before the surgery, respectively, reversed allodynia in the rat (“Brennan”) model of post-surgical pain for 5 days^22^.

Intraoperative AboBoNT-A treatment was also associated with reduced pain-related distress behaviors in pigs (wound guarding, reduced social interaction and withdrawal when approached) suggesting its efficacy in non-evoked, resting pain. These effects of aboBoNT-A preceded those on mechanical sensitivity, as they were detected 6 h after surgery. The effects of BoNT-A on measures of non-evoked, resting pain have been described in other models of post-surgical pain. For example, in rats, intraplantar or intrathecal injection of onaBoNT-A significantly reduced the cumulative post-surgical pain score–a measure of non-evoked pain based on the position of the operated paw in relation to the cage floor^22^. Also, aboBoNT-A reduced non-evoked postoperative pain and need for rescue analgesia in dogs undergoing radical bilateral mastectomy^23^. Overall, the analgesic efficacy of aboBoNT-A in the post-surgical pain pig model is well aligned with few examples of its use in the clinic. For example, in a retrospective clinical study conducted in patients undergoing mastectomy, 100 U of onaBoNT-A administered via intra-operative muscle infiltration route, significantly reduced immediate postoperative pain, the need for rescue analgesia and pain associated with the expander breast reconstruction^24^. In a multicenter prospective clinical study conducted in pediatric patients undergoing lower extremity limb lengthening and deformity correction, intramuscular BoNT-A injection immediately before surgery reduced post-operative pain for 4 days, reduced the need for rescue analgesia and improved quality of life^25^.

Here, for the first time, we provide evidence that peripherally administered BoNT-A reduces pain-related anxiety- or depression-like reactivity in an animal model. AboBoNT-A effects were seen in some distress behaviors (withdrawal when approached, reduced sociability) as well as in the outcome of the Approaching test. Specifically, intraoperative aboBoNT-A injection shortened approaching responses starting 6 h after treatment, resolving behavioral conflict, normalizing social bonding and enabling further socialization as detected in further shortening of approach latencies in aboBoNT-A-injected animals compared with the saline-injected group.

Considering the importance of social interaction for the animal, behavioral changes observed following aboBoNT-A treatment may be considered a surrogate for improved wellbeing in humans. Supporting translational validity of these data, there is growing clinical evidence of the antidepressant-like activity of BoNT-A, although whether it is a direct or indirect effect remains to be investigated. For example, in a randomized, double-blind, crossover clinical study and in a subsequent pooled analysis, BoNT-A injected in the forehead region improved symptoms of depression in patients with major depressive disorder^26,27^.

Importantly, a recent review of 40,000 post marketing safety reports in the FDA Adverse Event Reporting System showed that patients who received BoNT injections for symptomatic treatment in a range of conditions (spasticity, spasms, hyperhidrosis, migraine) and for esthetics, reported a significantly lower number of depression reports regardless of the injection site, compared with patients undergoing different treatments for the same conditions^28^. These findings suggest that while the facial feedback and emotional proprioception hypothesis of the antidepressant activity of BoNT^29^ if plausible, anxiolytic or antidepressant effects of BoNT are mediated by more complex central mechanisms, involving the brain (see below). We acknowledge that there are several differences in experimental variables between the current translational study and the clinical studies reviewed by Makunts et al.^28^. However, we hypothesize that the neuroanatomical substrate mediating anxiolytic and/or antidepressant-like effects of aboBoNT-A associated with ongoing pain in pigs as well as those detected in human patients, are fundamentally the same.

The effects of aboBoNT-A in post-surgical pain are likely to be prolonged. In the current study, we saw signs of enduring activity of aboBoNT-A in pain-related responses. As in the current model, pain-related readouts show natural recovery in 7–8 days following surgery (Meilin, personal communication), the model is not suitable for the assessment of the maximal duration of aboBoNT-A’s activity in post-surgical pain. As in the clinic, analgesic efficacy of BoNT-A across multiple chronic pain conditions, including migraine, lasts between 3 to 6 months^30^, we can speculate that similar duration of action can be expected in post-operative pain. Thus, in addition to acute pain, intraoperative aboBoNT-A treatment can reduce the likelihood of persistent or chronic post-surgical pain.

All doses of aboBoNT-A (100, 200 and 400 U) were well tolerated. AboBoNT-A-injected animals showed normal weight gain and locomotor activity as well as a complete lack of systemic or local adverse effects. The level of wound inflammation was low and similar across treatment groups and there was no treatment effect on wound healing or skin microvasculature. Thus, the effects of aboBoNT-A on pain-related behavioral responses described here were not confounded by adverse effects or by differences in wound inflammation, healing process or granulation tissue formation, suggesting that aboBoNT-A has a specific analgesic efficacy.

The peripheral sensory afferent nerve function is unlikely to be altered in aboBoNT-A-treated animals. Peripheral injections of BoNT-A do not alter normal nociceptive thresholds or acute nociceptive pain in both humans and animals^31–33^. In a recent study, we evaluated in vivo calcium imaging in the ipsilateral DRG neurons in mice exhibiting CFA-mediated inflammatory pain, that were treated with recombinant BoNT-A or vehicle^34^. While BoNT-A injections reduced mechanical allodynia (measured with von Frey filaments) in comparison to vehicle treatment, calcium fluctuations of BoNT-A and vehicle-treated animals in the DRG were similar^34^. This suggests that peripherally-administered BoNT-A does not change nociception-related signal transmission from the peripheral sensory afferents to the DRG. Based on these findings we concluded that spinal, rather than peripheral, mechanisms mediate analgesic efficacy of BoNT-A in inflammatory pain.

In the present study we used immunohistochemical detection of cleaved SNAP25 as a pharmacodynamic biomarker. Specifically, we assessed enzymatic activity of aboBoNT-A, in order to identify anatomical sites of its activity, as performed previously in rodent studies^35–39^. This is the first study where cleaved SNAP25 was investigated following a specific, intradermal administration of BoNT-A. We harvested the tissue from experimental animals at the end of the study (Day 6), making it impossible to investigate earlier time points, which is a limitation of IHC analysis.

The total, uncleaved SNAP25 was clearly present in the skin adjacent to the injection area, specifically in nerve endings around small arteries, in arrector pili muscles and in nerves in the deep and superficial dermis in both aboBoNT-A- and saline-injected animals. Unlike total SNAP25, cleaved SNAP25 was absent from these structures in aboBoNT-A-injected animals. This suggests that analgesic activity of aboBoNT-A, at least near the end of the study, did not require interruption in the release of pain neuromediators in the skin and therefore, was not dependent on peripheral mechanisms. Cleaved SNAP25 was also absent in the DRG of aboBoNT-A-injected animals, suggesting that analgesic efficacy of aboBoNT-A, at least following its peripheral administration, does not require its action in the DRG and modulation of its nociception-related activity. The enzymatic activity of aboBoNT-A was detected in the central nervous system, as indicated by the presence of cleaved SNAP25 in the ipsilateral and contralateral dorsal horns of the lumbar spinal cord of aboBoNT-A-injected animals and its absence from these sites in saline-injected controls. Indeed, cleaved SNAP25 staining was observed in almost all L5-L6 spinal cord sections, which receives the somatosensory innervation from the incision area of the skin and located in the lateral part of the substantia gelatinosa of Rolando in the ipsilateral dorsal horn. A minimal cleaved SNAP25 staining was noted in the same regions of the contralateral dorsal horn of aboBoNT-A-injected, but not saline-injected animals. The contribution of the injected area in distribution of cleaved SNAP25 in the lumbar spinal cord was further supported by its anatomical rostro-caudal distribution, as it was weaker at L1-L2 and L3-L4 levels. Cleaved SNAP25 immunolabelling was absent around motor neurons in ventral horns on both ipsilateral or contralateral sides. This suggests that intradermally injected aboBoNT-A taken up specifically by sensory neuronal terminals around the incision undergoes retrograde axonal transport from the injected dermis to the upper lamina of the dorsal horn most likely via the DRGs^40,41^. In rats, intramuscular injections of a low dose of onaBoNT-A (5 U/kg), or high doses of onaBoNT-A administered via intramuscular, subcutaneous or intra-nerve routes result in cleaved SNAP25 in the ipsilateral dorsal horn of the spinal cord^38^. Unlike the pattern of cleaved SNAP25 distribution in rodents^42^, there was no cleaved SNAP25 in the ventral spinal cord, suggesting that in pigs, intradermally injected aboBoNT-A was taken up only by sensory neurons and not by motor neurons. Such anatomical specificity in the uptake of BoNT is virtually impossible to achieve in rodent species, where it is binds to both sensory and motor neurons.

Spinal microglia and astrocytes have been known to react to a range of peripheral insults, including incision, and are known to contribute to post-surgical pain^43–46^. We assessed GFAP and Iba1 as markers of astrocyte^47^ and microglial^48,49^ activation, respectively. We can speculate that patterns GFAP and Iba1 in the lumbar spinal cord of saline-treated animals are linked to non-evoked, resting pain. The specificity in the rostral-caudal distribution of GFAP (L3-L4 and L5-L6) and especially of Iba1 (L5-L6 level only) links this pattern to the site of sensory stimulation coming from the incision, whereas the bilateral and diffuse nature of immunolabelling points at potential descending modulatory mechanisms. In addition, an intense focal induction of Iba1 in the lateral part of the substantia gelatinosa ipsilateral to the incision, suggests that there is an on-going, focal, pain-related sensory stimulation of microglia from the site of the incision located in the substantia gelatinosa ipsilateral to the incision.

Unilateral administration of aboBoNT-A intraoperatively resulted in a robust bilateral reduction in the GFAP immunostaining and milder bilateral reduction in the Iba1 immunolabelling. Based on the bilateral and diffuse pattern of these reductions, we hypothesize that the analgesic efficacy of aboBoNT-A involves, in part, central pain mechanisms. Reductions in pain-related anxiety and depression-like reactivity in aboBoNT-A-treated animals also points at the involvement of brain mechanisms involved in pain and those involved in anxiety/depression-like reactivity. Overall, we hypothesize that the analgesic efficacy of aboBoNT-A in post-surgical pain is mediated, in part, by modulation of spinal neuronal activity and by the reduction of astrocyte and microglial activation in the spinal cord combined with changes in brain pain processing. These findings confirm and expand earlier findings on bilateral effects of BoNT following unilateral administration. Our own work^50,51^ and those by others^52^ showed that unilateral injections of aboBoNT resulted in bilateral reduction in mechanical hyperalgesia in rodent models of polyneuropathic pain, mirror pain or bilateral inflammatory hyperalgesia.

We found no difference in CGRP and substance P immunolabelling between aboBoNT-A- and saline-injected animals both in the lumbar spinal cord and DRG. On the one hand, in an in vitro study BoNT-A blocked KCl-evoked release of CGRP and SP in primary sensory neuronal cultures^53,54^. On the other hand, according to Ishida et al.^55^, mice deficient in the α isoform of CGRP and wild-type controls exhibited similar mechanical sensitivity in the von Frey test following plantar incision. Thus, CGRP may not play a major regulatory role in post-surgical pain. This conclusion needs to be taken with caution, as additional studies with more sensitive methods of analysis are needed to quantify changes in concentrations of CGRP following aboBoNT-A treatment.

Unlike CGRP, there is evidence of the role of substance P in post-surgical pain. For example, mice with a deletion of preprotachykinin A gene (which codes for substance P) displayed reduced incision-induced mechanical allodynia and heat hyperalgesia in comparison to wild-type controls^46^. Also, according to Chen et al.^56^ SMIR-stimulated increase in SP concentration in DRG can be detected both 14 and 28 days after surgery. Thus, analgesic activity of aboBoNT-A in the pig appears to bypass the spinal substance P. Alternatively, the effect of aboBoNT-A on spinal substance P is present at earlier time-points (before 6 days) than measured in this study. Additional studies are needed to investigate these hypotheses.

It is a limitation of the current study that aboBoNT-A was administered at a single timepoint, intraoperatively, and only using intradermal route of administration. A systematic head to head assessment of subcutaneous, intramuscular and intradermal routes of aboBoNT-A administration in the current model is needed, in order to optimize the injection conditions before the approach is evaluated in a clinical study. Similarly, the timing of aboBoNT-A injection may influence the latency of onset of analgesic efficacy of BoNT-A in post-surgical pain. We can speculate that pre-emptive administration of aboBoNT-A before the surgery can result in an earlier onset of its analgesic efficacy. Systematic evaluation of the timing of the injection in the current model could provide another valuable information on optimization of aboBoNT-A injection conditions for the clinic. Another limitation of the study is that the IHC analysis of cleaved SNAP25 and pain-related biomarkers was performed only at a single time-point at the end of the study (Day 6) and did not include naïve, unoperated animals. A time course evaluation of the IHC biomarkers and inclusion of unoperated and untreated controls would have strengthened the behavioral results.

In conclusion, in a pig model of post-surgical pain, a single intraoperative treatment of aboBoNT-A via intradermal route of administration resulted in effective and prolonged relief of evoked and non-evoked pain. In addition, effective reduction of pain-associated anxiety/depression-like reactivity was also demonstrated. The analgesic effect of aboBoNT-A does not seem to require peripheral activity. It appears to be driven largely by its action on spinal neurons, astrocytes and microglia and may involve changes in pain processing in the brain. These data support further studies using the current model in order to optimize aboBoNT-A injection conditions and subsequent clinical evaluation of aboBoNT-A as a novel option for the effective, multimodal relief of post-surgical pain.

## Materials and methods

### Study design

The study was conducted with five treatment groups (N = 30; n = 6 per group). The sample size was decided based on multiple previous studies evaluating efficacy of analgesic drugs in this model^8^. The primary outcome measure that was used to determine the sample size was mechanical sensitivity measured in the von Frey test (see below;^8^). Animals were habituated before surgery (performed on Day 0) as described previously^8^. We performed SMIR surgery instead of just a full-skin incision one in order to obtain more pronounced signs of evoked and non-evoked pain and to better mimic operations performed in the clinic^11^. Following incision closure immediately after surgery, pigs received intradermal injections of either aboBoNT-A 100, 200 or 400 U/animal or saline as a negative control. Liposomal extended-release bupivacaine, used as a positive control, was infiltrated into the wound. Three animals were housed per pen, and all received the same treatment. All animals were assessed by the same investigator, blinded to treatment allocation throughout the study. Behavioral assessment involved a standard battery of tests which included von Frey (for mechanical sensitivity), Approaching, and Distress Behavior Score (DBS) tests, performed in the home pen (see ‘Assessments’ and Table S3). Mechanical sensitivity and guarding behavior are considered surrogates for evoked, movement-related pain and non-evoked, resting pain, respectively, which are seen in both human patients after surgery^12,13^ and in a rodent model^14^.

The Approaching then the DBS test were performed on all three animals housed in a pen simultaneously, followed by the von Frey test performed individually. Assessments were performed the day before surgery (Day -1) to obtain a baseline in each measure and on Days 0 to 6 (Table S3). On the day of the surgery (Day 0), full and shortened battery (DBS and von Frey testing only) were performed twice each (at 2 h and 6 h, and at 1 h and 4 h, respectively post-surgery; Table S3). From Day 1, behavioral tests were performed in the morning, at approximately 8:30 am (at least 1 h after the morning feed). The Open field test was performed on Days -1 (habituation) and 3. Wound inflammation was evaluated daily. Animals were weighed on Days -5 and 5 at the end of behavioral testing, and before surgery and testing on Day 0.

On Day 6, after the final Approaching test and incision inflammation scoring, animals were euthanized with an intraperitoneal injection of sodium pentobarbitone (> 100 mg/kg). Tissues were then collected from animals in the aboBoNT-A 400 U and saline groups (n = 3/group), for immunohistochemical (IHC) analysis. We collected tissues from the animals treated with only the highest dose of aboBoNT-A (400 U) because, on Day 5, the last day of the full behavioral assessment, pain-related behavioral responses in animals treated at 100, 200 or 400 U of aboBoNT-A were at their maximum and virtually identical. Therefore, the tissue collected from 400 U aboBoNT-A-treated group was considered to be a good representative for the “analgesic” responses shown by all aboBoNT-treated animals by the end of the study.

Experimental procedures were reviewed and approved by the MD Biosciences Institutional Animal Care and Use Committee (Ness Ziona, Israel). Studies were performed in full compliance with guidelines of the Israel National Ethics Committee, Committee for Research and Ethical Issues of the International Association for the Study of Pain^57^, ARRIVE guidelines and the US National Research Council Guide for the Care and Use of Laboratory Animals.

### Animals

Young (8–10 weeks old), male Danish Landrace x Large White cross-bred castrated pigs, were provided by Lahav Labs (Negev, Israel). All animals were drug or test naïve and in good health as confirmed by an attending veterinarian. Animals were acclimated for 5 days before the experiment. Throughout the acclimatization and testing period animals were housed in smooth-walled pens (140 × 240 cm), three per pen and maintained on a 12 h light/dark cycle (lights on from 07:00 to 19:00 h) under a constant temperature (21 ± 2 °C) and humidity (55 ± 5%).

Food (Dry Sows; Ct # 5420; Milobar; Oshrat, Israel) was provided twice daily and water ad libitum. Animals were provided enrichment to root and chew on, and were given a unique identification ear mark in the form of a four-digit number. Animals weighed between 11 and 13 kg at the start of the habituation period.

### Surgery

The anesthesia and the surgery were performed as described previously^8^. In the morning of the day of the surgery (Day 0), pigs were anesthetized with a 5% isoflurane/oxygen (2–3 L/min) mixture with the aid of anesthetic facemask (Stephan Akzent Color, Gackenbach, Germany). The anesthesia permitted relatively quick recovery for the assessment of behavioral responsiveness 1 h after the surgery/treatment. An incision was made on the left side of the lower back, approximately 3 cm lateral and parallel to the spine (Fig. S5). Before administering the incision, the area of the low back was scrubbed with antiseptic liquid (Polydine® solution, Dr. Fischer laboratories, Bnei Brak, Israel) and wiped with 70% ethanol. A 7-cm long, full skin thickness incision was made, including the fascia and the underlying muscle (gluteus medius) which was retracted to perform the incision. A sterile cover, disinfected with an antiseptic liquid (3% synthomycine), was placed around the incisional area. The fascia was sutured with 3–0 vicryl thread, while the skin was sutured with 3–0 silk thread (Assault UK Ltd., West Yorkshire, UK) using continuous suturing methods. Following the incision closure, animals received study treatment (described below) and an intramuscular injection of an antibiotic (10% w/v Marbocyl®; Vetoquinol UK Ltd., Buckingham, UK) into the neck. The animals were then returned to their pens for recovery and observation.

### Drug administration

AboBoNT-A was provided by Ipsen Limited (Wrexham, UK). Each vial containing 500 U of purified *C. botulinum* type A neurotoxin complex was reconstituted in saline (0.9% sodium chloride) to obtain the final concentration. AboBoNT-A (100, 200 or 400 U/pig) or saline were administered in 2 mL total volume split into 10 intradermal injections (0.2 mL/site) around the incision (Fig. S5) using 30 G needles attached to 1-mL syringes (BD Micro Fine Plus; Becton Dickinson, Plymouth, UK). Liposomal extended-release bupivacaine (EXPAREL) was provided as an injectable suspension for infiltration (266 mg/20 mL) by Pacira Pharmaceuticals, Inc., (Parsippany, NY, USA). Liposomal bupivacaine (26.6 mg/pig) was infiltrated in the incision as a 2-mL fixed volume using 21-G needle attached to 5-mL syringe.

### Assessments

#### von Frey test

Evaluation of mechanical sensitivity with von Frey filaments was conducted as described previously^8^. Von Frey filaments (Ugo Basile, Gemonio, Italy), which ranged from 1.0 to 60.0 g (Table S4), were applied approximately 0.5 cm proximal to the incision line three times with interval of 5–10 s. Withdrawal reaction was considered if the animal moved away from the stimulus or twisted its flank; the accompanying vigorous tail wiggling was not considered a withdrawal reaction when independent. The von Frey test was performed by a dedicated investigator previously familiarized with the animal, while they were fed. If withdrawal was not achieved, a thicker filament was applied. If withdrawal by the animal from the filament did occur, a thinner filament was applied. Animals were pre-exposed to the von Frey test two days before the surgery (Day -2) to habituate them to the procedure and one day before the surgery (Day -1) for WF baseline. Animals were excluded from the study if their WF responses at baseline were below 26 g.

#### DBS test

The DBS test was performed to measure non-evoked, resting pain after surgery^8^. Expression of 14 behaviors, divided into seven categories, were monitored. These behaviors are either exhibited by normal, intact animals (behaviors with the score 0) or are specific to post-surgical period (behaviors with the score 1; Table S5). The total DBS is the sum of the scores on each category and can range from 0 (normal) to 7 (severely distressed). Assessment of DBSs were not performed in a particular order, as behaviors were recorded as exhibited by the animal. The DBS test lasted approximately 3 min.

#### Approaching test

The Approaching test was used to measure non-evoked pain-related anxiety- or depression-like reactivity. The basis of the Approaching test is a social bond that young pigs establish with their caregiver as a result of their daily interactions^58^. When the familiar caregiver enters their home pen, young, habituated animals are quick to approach them, whereas the young pigs are slow to approach unfamiliar individuals. Following SMIR, however, seeking social interaction with the caregiver is replaced with a behavioral conflict between approach and avoidance responses causing approach latencies to increase. We measured the latency (in seconds) to approach the investigator entering their home-pen using a cut-off time of 180 s.

### Open field test

The Open field test was performed as previously described^59^. Briefly, animals were placed individually into a rectangular arena (2.5 × 4.7 × 1.6 m) and monitored for locomotor activity for 5 min with a CCTV camera. The data were analyzed with AnyMaze software (Stoelting Co, Dublin, Ireland). The total distance travelled (m), speed of locomotion (m/s) and the percentage of time spent in the center of the area were quantified.

### Wound inflammation

Wound inflammation was scored based on two categories: redness (0- normal; 1- slight redness at the area of the incision; 2- spread redness) and swelling (0, no swelling; 1, slight swelling; 2, pronounced swelling). The final score for each animal is the sum of the scores in each category (max score = 4). The wound inflammation was scored once daily on Days 1 to 6.

### Histopathology and immunohistochemistry

#### Tissue sampling

The entire cutaneous incision site (9 × 2 × 1 cm) and the entire lumbar vertebral column (with the spinal cord and DRGs) were collected from the animals injected with 400 U aboBoNT-A or saline (n = 3/group). The tissues were fixed in 4% formalin for 48 h. For the skin, a transversal section was made at the middle of the cutaneous incision. The lumbar spinal cord was isolated and separated in 3 equal parts (L1-L2, L3-L4 and L5-L6). Each part was sectioned in about 10 tissue sections of 5 mm each (i.e. 10 spinal cord sections per cassette). Dorsal root ganglia emerging from L4/L5/L6 were sampled on both sides. All tissue samples were processed to paraffin blocks. Four micrometer sections were stained with hematoxylin and eosin (H&E) and paraffin slides were produced for immunohistochemical (IHC) analyses.

Wound healing was evaluated by a pathologist (SL) using H&E sections. CD31 (ab28364; Abcam) IHC and image analyses on full tissue sections (Nanozoomer, Hamamatsu, Japan) at the level of the deep granulation tissue (layer 1), superficial granulation tissue (layer 2) and in the surrounding superficial dermis (adjacent to healing part/layer 3; Fig. S3) were used to quantify vessel density (number of blood vessels/mm^2^) and size (blood vessel surface area) with the aid of the Object colocalization module (HALO™ system, Indica labs, Albuquerque, NM, USA).

### IHC analyses of synaptosomal-associated protein 25 kD (SNAP25) and pain-related biomarkers

The IHC analyses were performed using a standard streptavidin–biotin–peroxidase procedure and specific heat mediated antigen retrieval methods. Endogenous peroxidase was blocked for 10 min in a 3% hydrogen peroxide solution in a tris-buffered saline (TBS) buffer. The sections were incubated with a primary rabbit polyclonal antibody (EF14007, noncommercial; Ipsen Innovation, Les Ulis, France) which is specific for the BoNT-A-cleaved form of SNAP25^42^. In this study, we confirmed the specificity of our antibody (EF14007; noncommercial antibody) for cleaved SNAP25^42^. Our antibody recognizes cleaved SNAP25 in human and rodent tissues injected with BoNT-A^42,60^. In the pig, cleaved SNAP25 was only detected in aboBoNT-A-injected animals and not in saline-injected controls, suggesting that the antibody detects specifically the cleaved SNAP25 also in pigs. After washing the slides with TBS, the sections were incubated with a biotinylated secondary antibody for 10–15 min (anti-Rabbit IgG). Sections were then washed with TBS and incubated for 30 min with an amplification system (streptavidin–biotin–peroxidase, ABC vector) for 30 min. After a wash in TBS, sections were incubated for 5–10 min with a solution of 0.02% diaminobenzidine containing 0.01% hydrogen peroxide. An antibody directed against the N-terminal part of SNAP25 (SYSY 111011, aa 20–40) was used in the same condition to detect total SNAP25 in tissues. Counterstaining was performed using aqueous hematoxylin. The presence of cleaved SNAP25 in the spinal cord was quantified by counting the number of cleaved SNAP25 positive spinal cord sections on each slide.

IHC was also used to investigate glial fibrillary acidic protein (GFAP, Dako Z0334), ionized calcium-binding adaptor protein-1 (Iba1; ab178847; Abcam, Paris, France), substance P (ab67006; Abcam, Paris, France), and calcitonin gene-related peptide (CGRP, C8198 (Sigma Aldrich; Saint Quentin Fallavier, France). GFAP and Iba1 immunostaining were quantified by image analyses (HALO, percentage of positive pixels in the entire ipsilateral dorsal horns (GFAP) or in the focal, high density area in the same dorsal horn (Iba1, Fig. 6, arrowheads). For other markers, i.e. substance P and CGRP, immunostainings were quantified as minimal (grade 1), moderate (grade 2) or marked (grade 3). All slides were evaluated by a board-certified veterinary pathologist (SL) using a light microscope (Olympus BX41).

### Data analysis

Mechanical sensitivity data (von Frey test) were first transformed to quoted stimulus by a log transformation of the gram units multiplied by 10,000. The analysis includes a full factorial repeated measure mixed linear model. The fixed effects include treatment, time and their interaction while the random effects were estimated for animal nested in treatment and time. Tukey post hoc test was derived to compare any group at any time with any other (including itself over time). The approaching time was treated as a continuous variable, and therefore, analyzed using a full factorial repeated measure mixed linear model with fixed effects and random coefficients identical to the mechanical sensitivity analysis. Analyses were performed using JMP®, Version 14 (SAS Institute Inc., Cary, NC, USA). The DBS, body weight, incision score and open field test data were analyzed using one-way ANOVA followed by Tukey post-hoc test.

Statistical analysis of the blood vessel density data was completed using repeated measures ANOVA. To compare each layer a Student t-test was used. For the blood vessel size evaluation, vessels were classified in 8 quantiles (from “small –", to “large +  + ” in saline injected animals). Data from AboBoNT-A injected animals were compared to saline injected animals (controls) using a Fischer exact test for the 3 layers. Two-way ANOVA was used to compare the staining intensity of GFAP and Iba1 in the different levels of the spinal cord on 2 tissue sections per paraffin block (3 animals/group, 6 tissue sections/group).

## Supplementary Information

Supplementary Information.

## Data Availability

The data that support the findings of this study are available from Ipsen but restrictions apply to the availability of these data, which were used under license for the current study, and so are not publicly available.
