# Supplementary material for: Intraoperative abobotulinumtoxinA alleviates pain after surgery and improves general wellness in a translational animal model
Source: Sci Rep. 2022 Dec 13;12:21555. doi: 10.1038/s41598-022-25002-x (PMC9747791; doi:10.1038/s41598-022-25002-x)
Supplement: Supplementary file 1 — Supplementary Information. [file 41598_2022_25002_MOESM1_ESM.docx]

**Supplementary Materials**

**Supplementary Figures**


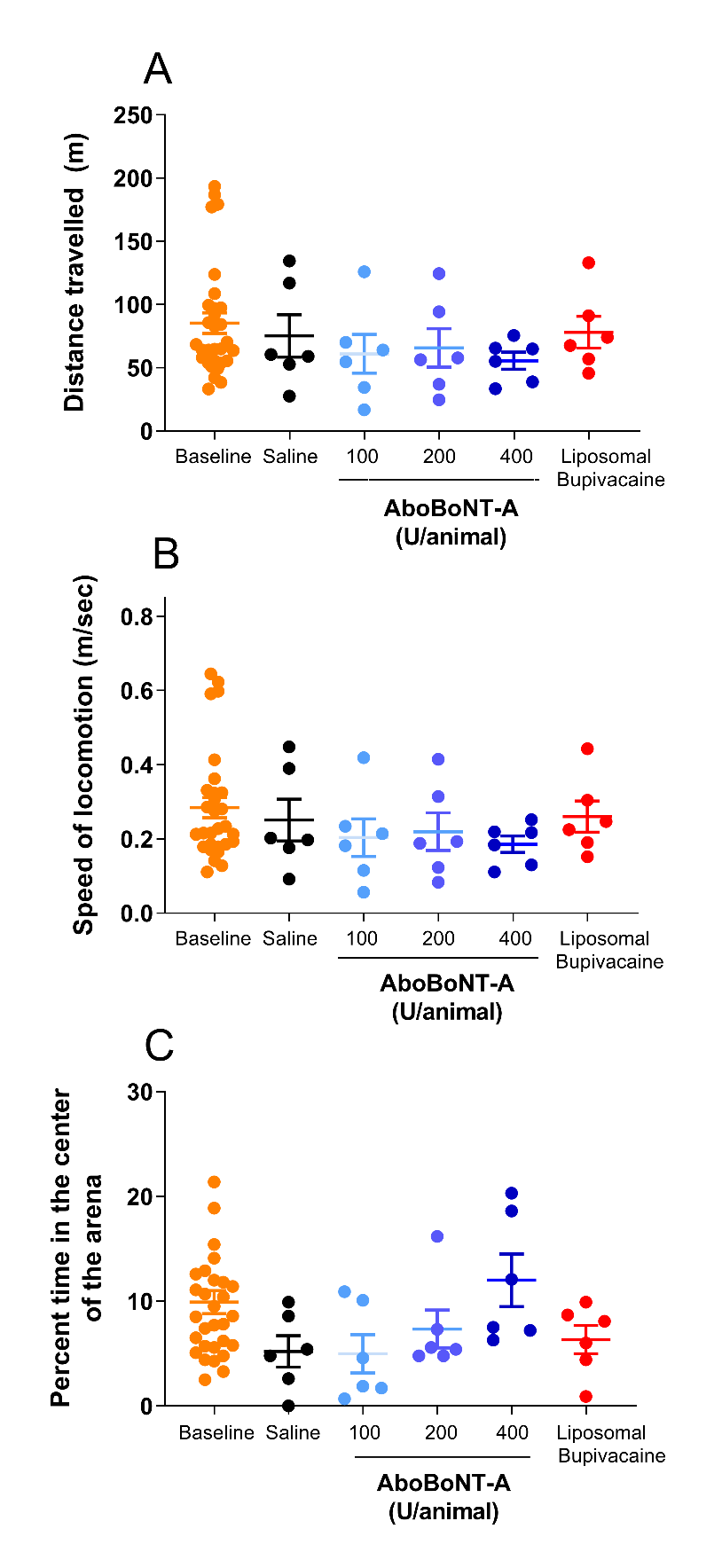


**Figure S1.** **Spontaneous locomotor activity in the Open field test in aboBoNT-A-, saline-, or liposomal bupivacaine-treated animals.** Animals that received a full-skin and muscle incision and retraction surgery (Day 0), followed by intraoperative treatment with either aboBoNT-A (100 U, 200 U, or 400 U), saline, or liposomal bupivacaine (n=6/group) were evaluated in the Open field test for the distance travelled (A), speed of locomotion (B) and percent time spent in the central zone (C).

AboBoNT-A, abobotulinumtoxinA; U, unit

**Figure S2.** **Effects of aboBoNT-A, saline, and liposomal bupivacaine on the number of animals** **with wound redness**. Animals that received a full-skin and muscle incision and retraction surgery (Day 0), followed by intraoperative treatment with either aboBoNT-A (100 U, 200 U, or 400 U), saline or liposomal bupivacaine (n=6/group) were evaluated for wound redness on days (D) 1-6.

AboBoNT-A, abobotulinumtoxinA; U, units, D, day


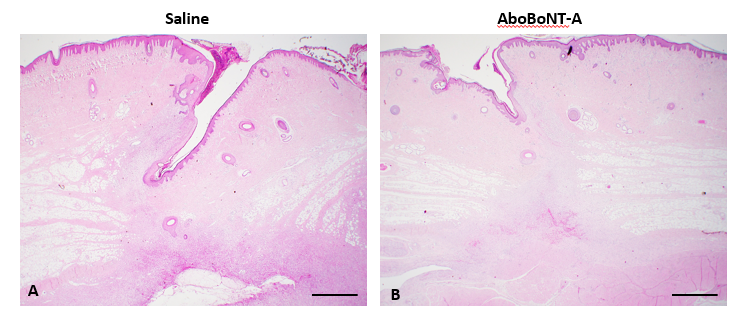


**Figure S3.** **Wound healing and inflammation in animals treated with aboBoNT-A or saline.** Photomicrographs depict cross-sectional view of the skin around the incision area as well as inflammatory reactions (H&E sections) on Day 6 collected from representative animals that received a full-skin and muscle incision and retraction surgery (Day 0), followed by intraoperative treatment with either aboBoNT-A (400 U) or saline. Scale bars: 1 mm

AboBoNT-A, abobotulinumtoxinA; U, units


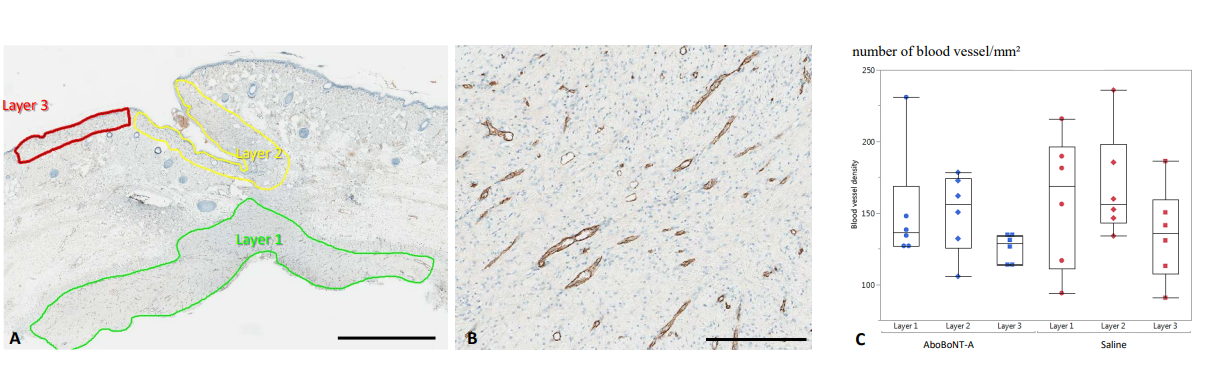


**Figure S4**. **Skin blood vessels around incision area in aboBoNT-A and saline-treated animals.** Immunohistochemical labelling of CD31 in a cross-section from the dermis around the incision area (A) and in granulation tissue in layer 1 (B) from representative animals that received a full-skin and muscle incision and retraction surgery (Day 0), followed by intraoperative treatment with aboBoNT-A (400 U). Panel C shows whisker boxes plots of the blood vessel density (number of blood vessel/mm^2^) performed in layers 1, 2 and 3 in representative animals (n=3/group) treated with either aboBoNT-A (blue symbols) or saline (red symbols; n=2 sections per animal). Scale bars: 3 mm (A), 1 mm (B).

AboBoNT-A, abobotulinumtoxinA; U, units


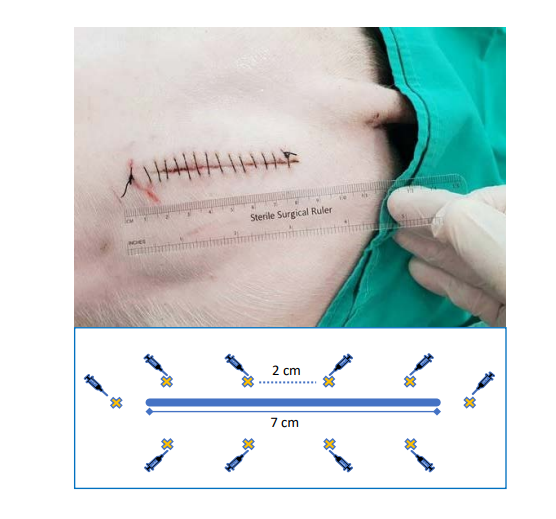


**Figure S5.** **Location of aboBoNT-A or saline injections.** Photo of the incision on the lower back in a representative animal and schematic representation of intradermal aboBoNT-A or saline injection sites (crosses) distributed around the incision (blue line).

AboBoNT-A, abobotulinumtoxinA.

**Supplementary Tables**

| **Treatments** | **Mean, kg (SEM)** | | | **Mean change from baseline, % (SEM)** | |
| --- | --- | --- | --- | --- | --- |
|  | **Day -5** | **Day 0** | **Day 5** | **Day 0** | **Day 5** |
| Saline | 11.63±0.15 | 12.59±0.21 | 14.45±0.45 | 8.33±2.36 | 24.3±4.24 |
| AboBoNT-A 100 U | 11.17±0.35 | 12.06±0.22 | 14.53±0.44 | 8.40±3.23 | 30.49±4.57 |
| AboBoNT-A 200 U | 10.88±0.30 | 11.73±0.40 | 14.63±0.67 | 7.85±3.17 | 34.47±5.61 |
| AboBoNT-A 400 U | 11.32±0.22 | 12.02±0.22 | 14.48±0.55 | 6.24±1.40 | 27.93±3.82 |
| Liposomal bupivacaine | 11.27±0.38 | 11.99±0.36 | 14.24±0.55 | 6.57±1.84 | 26.74±4.53 |

**Table S1**. **Body weight change in aboBoNT-A, saline- and liposomal bupivacaine-treated animals.** Absolute body weight (Mean ± SEM) and change from baseline in pigs five days before a full-skin and muscle incision and retraction surgery (Day -5), on the day of the surgery (Day 0) and 6 days after the surgery and intraoperative treatment (n=6/group).

AboBoNT-A, abobotulinumtoxinA; SEM, standard error of the mean; U, unit.

| **Layers** | **Treatment** | **Small**  **--** | **Small**  **-** | **Medium**  **--** | **Medium**  **-** | **Medium**  **+** | **Medium**  **++** | **Large +** | **Large ++** |
| --- | --- | --- | --- | --- | --- | --- | --- | --- | --- |
| Layer 1 | AboBoNT-A | 12,91 | 12,64 | 11,76 | 12,39 | 12,10 | 12,41 | 12,94 | 12,84 |
|  | Saline | 12,87 | 12,27 | 12,31 | 12,55 | 12,43 | 12,61 | 12,46 | 12,50 |
| Layer 2 | AboBoNT-A | 10,81 | 11,65 | 12,08 | 11,86 | 13,35 | 11,02 | 15,68 | 13,56 |
|  | Saline | 13,41 | 16,09 | 12,26 | 18,39 | 14,18 | 9,58 | 10,73 | 5,36 |
| Layer 3 | AboBoNT-A | 11,83 | 15,05 | 11,83 | 14,52 | 12,37 | 11,83 | 11,29 | 11,29 |
|  | Saline | 9,68 | 14,19 | 9,03 | 14,84 | 12,26 | 12,26 | 9,68 | 18,06 |

**Table S2: Blood vessels in the skin of aboBoNT-A- and saline-treated animals.** Percentage of Small -- to Large ++ blood vessels in the 3 layers of the skin around the incision site analyzed using image analysis and CD31 immunohistochemistry. Tissues were collected from representative animals (n=3/group) that were treated with either aboBoNT-A or saline (n=2 sections per animal).

AboBoNT-A, abobotulinumtoxinA

| **Study part** | **Study Day** | **Procedures** |
| --- | --- | --- |
| Habituation period and baseline assessment | -5 | Body weight |
|  | -2 | von Frey test (habituation) |
|  | -1 | - Approaching test (baseline) - DBS test (baseline) - von Frey test (baseline) - Open field (habituation) |
| Surgery and treatment | 0 | Body weight  *Surgery & treatment*   \| **Time post treatment (Hours)** \| **Tests** \| \| --- \| --- \| \| 1 \| - DBS test - von Frey test \| \| 2 \| - Approaching test - DBS test - von Frey test \| \| 4 \| - DBS test - von Frey test \| \| 6 \| - Approaching test - DBS test - von Frey test \| |
| Follow up testing | 1–5 | - Approaching test - DBS test - von Frey test - Wound inflammation scoring - Open field test (Day 3) - Body weight (Day 5) |
| Termination | 6 | - Approaching test - Wound inflammation scoring - Tissue harvesting for analysis |

**Table S3. Schedule of tests**

DBS, Distress Behavior Score

|  | **Filament in use** | | | | | | | | | |
| --- | --- | --- | --- | --- | --- | --- | --- | --- | --- | --- |
| Size | 4.08 | 4.17 | 4.31 | 4.56 | 4.74 | 4.93 | 5.07 | 5.18 | 5.46 | 5.88 |
| Force (g) | 1.00 | 1.40 | 2.00 | 4.00 | 6.00 | 8.00 | 10.0 | 15.0 | 26.0 | 60.0 |

**Table S4.** **Range of sizes of von Frey filaments used in the study.**

| **Scoring category** | **Parameter** | **Score** |
| --- | --- | --- |
| Category 1. | Avoiding standing (lying down) | 1 |
|  | Standing | 0 |
| Category 2. | Avoiding walking | 1 |
|  | Walking | 0 |
| Category 3. | Guarding behavior (protecting the wound side while walking) | 1 |
|  | Acting normal | 0 |
| Category 4. | Moving away when approached by the investigator | 1 |
|  | Not moving away when approached by investigator | 0 |
| Category 5. | Restlessness | 1 |
|  | Normal | 0 |
| Category 6. | Staying in isolation from other animals | 1 |
|  | Staying together with other animals | 0 |
| Category 7. | High pitch/distress vocalization | 1 |
|  | Normal vocalization | 0 |

**Table S5.** Scoring categories in the DBS test to evaluate the presence of behaviors that can be expressed following surgery (score 1) or are typical for a normal, intact animal (score 0). DBS, Distress Behavior Score
